# Etiology of Central Nervous System Infections in a Rural Area of Nepal Using Molecular Approaches

**DOI:** 10.4269/ajtmh.18-0434

**Published:** 2019-06-03

**Authors:** Olof Säll, Sara Thulin Hedberg, Marita Neander, Sabina Tiwari, Lester Dornon, Rabin Bom, Nina Lagerqvist, Martin Sundqvist, Paula Mölling

**Affiliations:** 1 Department of Infectious Diseases, Faculty of Medicine and Health, Örebro University, Örebro, Sweden;; 2 Department of Laboratory Medicine, Clinical Microbiology, Faculty of Medicine and Health, Örebro University, Örebro, Sweden;; 3 United Mission Hospital Tansen, Tansen, Nepal;; 4 Public Health Agency of Sweden, Solna, Sweden

## Abstract

The etiology of infections of the central nervous system (CNS) in Nepal often remains unrecognized because of underdeveloped laboratory facilities. The aim of this study was to investigate the etiology of CNS infections in a rural area of Nepal using molecular methods. From November 2014 to February 2016, cerebrospinal fluid (CSF) was collected from 176 consecutive patients presenting at United Mission Hospital in Tansen, Nepal, with symptoms of possible CNS infection. After the CSF samples were stored and transported frozen, polymerase chain reaction (PCR) was performed in Sweden, targeting a total of 26 pathogens using the FilmArray^®^ ME panel (BioFire, bioMerieux, Salt Lake City, UT), the MeningoFinder^®^ 2SMART (PathoFinder, Maastricht, The Netherlands), and an in-house PCR test for dengue virus (DENV), Japanese encephalitis virus (JEV), and Nipah virus (NiV). The etiology could be determined in 23%. The bacteria detected were *Haemophilus influenzae* (*n* = 5), *Streptococcus pneumoniae* (*n* = 4), and *Neisseria meningitidis* (*n* = 1). The most common virus was enterovirus detected in eight samples, all during the monsoon season. Other viruses detected were cytomegalovirus (*n* = 6), varicella zoster virus (*n* = 5), Epstein–Barr virus (*n* = 3), herpes simplex virus (HSV) type 1 (HSV-1) (*n* = 3), HSV-2 (*n* = 3), human herpes virus (HHV) type 6 (HHV-6) (*n* = 3), and HHV-7 (*n* = 2). *Cryptococcus neoformans*/*gatti* was found in four samples. None of the samples were positive for DENV, JEV, or NiV. Of the patients, 67% had been exposed to antibiotics before lumbar puncture. In conclusion, the etiology could not be found in 77% of the samples, indicating that the commercial PCR panels used are not suitable in this setting. Future studies on the etiology of CNS infections in Nepal could include metagenomic techniques.

## INTRODUCTION

Infections in the central nervous system (CNS), which include meningitis and acute encephalitis syndrome (AES), are globally important causes of hospital admissions, significant mortality, and morbidity, including severe persistent neurological sequelae.^1,2^ A prompt start of adequate treatment is necessary in the more severe cases of bacterial meningitis and AES caused by herpes viruses to improve outcome.^1,2^ Central nervous system infections can be caused by a diverse spectrum of bacteria, viruses, parasites, and fungi. However, the causative agents cannot be determined on clinical symptoms alone as the symptoms are nonspecific.^3^ Therefore, microbiological testing is essential to determine the causing agents and to guide adequate antimicrobial treatment.^4,5^

In Nepal, the etiology of CNS infections is largely unknown, partly because of insufficient microbial laboratory facilities and lack of national surveillance programs. Previous hospital-based studies in the country have described a diverse etiology of CNS infections with the vaccine-preventable pathogens *Streptococcus pneumoniae*, *Haemophilus influenzae*, and Japanese encephalitis virus (JEV) being the most commonly found pathogens. However, in these studies, the etiology was unclear in 62–93% of the patients.^6–9^

Molecular diagnostic testing methods are first-line diagnostic tools in many high-income countries for the etiological diagnosis of CNS infections with the advantage of a rapid detection with high sensitivity compared with culture as well as exclusion of predefined pathogens. Molecular testing has shown to be useful to exclude bacterial CNS infections and, hence, shorten antibiotic treatment and hospital length of stay, as well as to detect pathogens after antibiotic treatment is initiated.^10,11^ These techniques could be of special importance for both clinical routine and surveillance purposes in settings where culture of the cerebrospinal fluid (CSF) is not available.^12^ A rapid molecular multiplex polymerase chain reaction (PCR) panel for CNS infections has been studied in low- and middle-income countries with a positivity rate of 10% in a general population^13,14^ and a higher detection rate in a cohort of HIV-infected patients.^15^

The aim of this study was to explore the etiology of community-acquired CNS infections at a regional hospital in Nepal using molecular methods.

## MATERIAL AND METHODS

### Setting.

**Figure 1.**
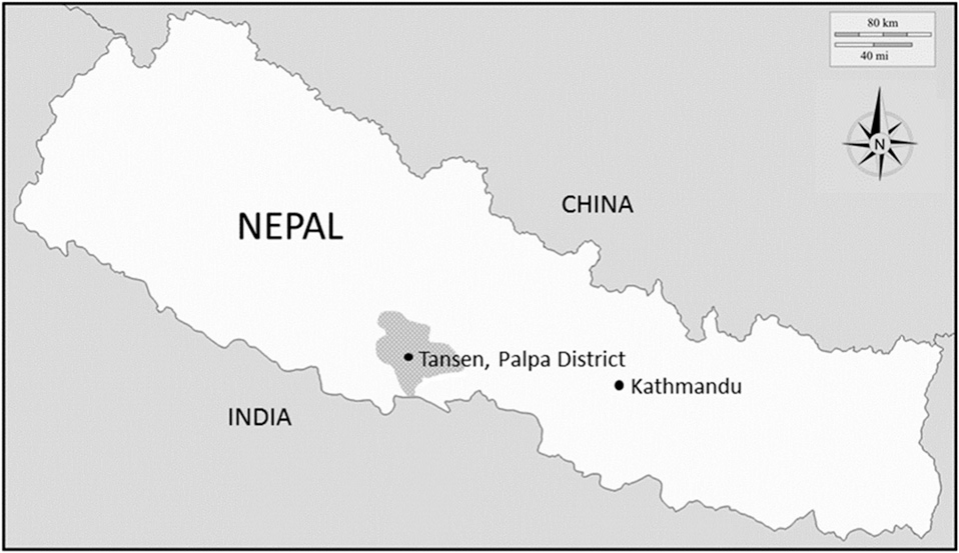
Map showing Nepal country borders; location of the study site; Tansen; and the surrounding gray-colored area of the patient catchment area.

United Mission Hospital Tansen (UMHT) is a nonprofit-making hospital, funded by patient fees and international support located in Tansen, Palpa district, at an altitude of 1,300 m in Nepal’s Western Development Region/Province No. 5 (Figure 1). Annually, the hospital serves 98,000 outpatients and 12,000 inpatients. The patients mainly come from Palpa and surrounding districts and also from the Indian states of Uttar Pradesh and Bihar.

The national immunization program in Nepal includes vaccines against measles, polio, and diseases caused by *H. influenzae* type b, *Mycobacterium tuberculosis*, measles, and polio, and according to official data, the national vaccination coverage in 2014–2015 was around 90%. During the study period, pneumococcal conjugate vaccine was partially introduced in the study area starting in January 2015.^16^

Seasonal outbreaks of meningitis and encephalitis occur mainly during the monsoon season from June to September with predominantly suspected viral cause.^8^ The incidence of JEV infections in the Palpa district during 2007–2015 has been estimated to be 0.5–0.9/100,000 population per year.^17^ Since 2006, JEV immunization has been implemented in the Nepalese districts with the highest JEV disease incidence, not including Palpa district.^18^

### Study design and participants.

This prospective observational study included participants consecutively between November 2014 and February 2016 with possible community-acquired CNS infection. Eligible for inclusion were patients at the UMHT presenting with one or more symptoms indicating CNS infection (fever, headache, neck stiffness, altered mental status, or other neurological manifestations) where lumbar puncture was performed as part of the routine investigations.

### Data collection.

Clinical and demographic data were collected on a patient record form. At the diagnostic lumbar puncture, 2–3 mL of CSF was collected, of which around 1 mL of CSF was used for the analyses at the UMHT laboratory (visual observation, white blood cell [WBC] count, including differentiation between polymorphonuclear and mononuclear cells, protein, and glucose). Another 1 mL of CSF was dedicated for the molecular testing, and these samples were stored at −20°C at the UMHT laboratory with daily temperature controls. Two months after sample collection was completed, all samples were transported on dry ice to the Department of Laboratory Medicine, Örebro University Hospital, Örebro, Sweden, and stored at −80°C pending further analysis. The median time interval from sample collection to transport to Sweden was 7 months. The PCR analyses for dengue virus (DENV), JEV, and Nipah virus (NiV) were performed at the Public Health Agency of Sweden, Solna, Sweden. All other molecular analyses were performed at the Department of Laboratory Medicine, Örebro University Hospital, Örebro, Sweden.

Inclusion was possible even if clinical, demographic, or laboratory data were incomplete. Tests for HIV antigen and antibodies and adenosine deaminase, and India ink staining were performed, if clinically indicated, and the results recorded but were not primarily a part of this study. No culturing, bacterial antigen tests, or serology on CSF were performed. Moreover, no serum/plasma samples for PCR and/or serology were available in this study.

### Polymerase chain reaction analysis.

#### FilmArray ME panel.

The FilmArray Meningitis/Encephalitis (ME) panel (BioFire, bioMerieux) is a multiplex PCR test,^19,20^ which includes six bacteria, seven viruses, and two fungi (Table 1).

**Table 1:** Results of analyses with FilmArray ME panel, MeningoFinder 2SMART, and in-house PCR targeted at JEV, DENV, and NiV, respectively, and compiled data of assessed number of samples of disease-causing pathogens detected; presented as total number and percentage (*n* = 176)

Pathogen	FilmArray ME panel	MeningoFinder 2SMART	In-house PCR	No of samples with pathogen found*
Bacteria				
*Borrelia burgdorferi* sensu lato*/Borrelia miyamatoi*	N/A†	0	N/A†	0
*Escherichia coli* K1	0	0	N/A†	0
*Haemophilus influenzae*‡	5 (3%)	4 (2%)	N/A†	5 (3%)
*Listeria monocytogenes*	0	0	N/A†	0
*Neisseria meningitidis*	1 (1%)	1 (1%)	N/A†	1 (1%)
*Staphylococcus aureus*	N/A†	0	N/A†	0
*Streptococcus agalactiae*	0	0	N/A†	0
*Streptococcus pneumoniae*‡	4 (2%)	4 (2%)	N/A†	4 (2%)
Viruses				
Cytomegalovirus	5 (3%)	1 (1%)	N/A†	6 (3%)
DENV	N/A†	N/A†	0	0
Ebstein–Barr virus	N/A†	3 (2%)	N/A†	3 (2%)
Enterovirus	8 (5%)	0	N/A†	8 (5%)
HSV type 1	3 (2%)	0	N/A†	3 (2%)
HSV type 2	0	3 (2%)	N/A†	3 (2%)
HHV type 6	3 (2%)	1 (1%)	N/A†	3 (2%)
HHV type 7	N/A†	2 (1%)	N/A†	2 (1%)
HHV type 8	N/A†	0	N/A†	0
Human parechovirus	0	0	N/A†	0
JEV	N/A†	N/A†	0	0
Morbillivirus	N/A†	0	N/A†	0
Mumps virus	N/A†	2 (1%)	N/A†	2 (1%)
NiV	N/A†	N/A†	0	0
Varicella zoster virus	5 (3%)	2 (1%)	N/A†	5 (3%)
Fungi				
*Cryptococcus neoformans*/*gattii*	4 (2%)§	3 (2%)/0§	N/A†	4 (2%)

DENV = dengue virus; HHV = human herpes virus; HSV = herpes simplex virus; JEV = Japanese encephalitis virus; NiV = Nipah virus; PCR = polymerase chain reaction.

* Assessment with all performed analyses taken into account.

† The pathogen not analyzed with the mentioned method.

‡ Please see supplement for detailed information.

§ In contrast to MeningoFinder 2SMART, the FilmArray ME panel does not specify whether *C. neoformans* or *C. gattii* was detected.

From the clinical samples, 200 μL of CSF was tested according to the manufacturer’s instructions. In brief, the FilmArray system consists of a fully automated system of integrated nucleic acid purification, reverse transcription, and nested multiplexed PCR. The FilmArray software performs automated result analysis where each target in a valid run is reported as “detected” or “not detected.” Whenever either of the included internal controls (an RNA process control or a nested PCR DNA control) fails, the software automatically provides a result of “invalid” for all panel analytes.

This study was conducted with a research-only version of the FilmArray ME panel that was identical to the final Food and Drug Administration cleared/CE-marked in vitro diagnostic version, with the exception that Epstein–Barr virus (EBV) is not available in the commercial product; therefore, positive results from EBV testing are not presented here.

#### DNA and RNA extraction.

For extraction of total nucleic acid (DNA and RNA), 200 μL of CSF was extracted using the QIAamp cador Pathogen Mini Kit using the QIAcube workstation (QIAGEN, Venlo, The Netherlands).^21^ If the sample volume was less than 200 μL (*n* = 45), sodium chloride was added to make up to 200 μL before extraction.

In samples where there was no remaining CSF after the FilmArray ME panel analysis (*n* = 20), extraction was performed on 200 μL of the CSF/lysis buffer mix prepared for the FilmArray analysis using the MagNA Pure Compact Nucleic Acid Isolation Kit I (Roche Diagnostics, Mannheim, Germany) and the MagNA Pure Compact system.

#### MeningoFinder 2SMART.

The MeningoFinder 2SMART multiplex PCR test (PathoFinder) includes nine bacteria, 12 virus, and two fungi (Table 1) and was tested according to the manufacturer’s instructions. The PCR started with pre-amplification, performed in a Veriti^®^ 96-well thermal cycler (Applied Biosystems, Thermo Fisher Scientific, Waltham, MA), and followed by two steps of separate real-time multiplex amplification using a Rotor-Gene Q instrument (QIAGEN). Detection of pathogens was performed using specific probes with unique melting points in three different channels. Melting curves were generated and manually assessed in relation to the interpretation rules provided by the manufacturer.

### Analysis of DENV, JEV, and NiV.

Dengue virus, JEV, and NiV one-step real-time reverse transcriptase PCR assays were carried out in 15-μL reactions containing 4 μL template, TaqMan Fast Virus 1-Step Master Mix, nuclease-free water, 0.2 μM probe, and each primer at 0.9 μM. Amplification and detection were performed in a StepOne Plus real-time PCR system (Applied Biosystems, Thermo Fisher) using the thermal cycling parameters described previously.^22^ The primers and probe used in the DENV real-time RT-PCR have been described elsewhere.^22^ Japanese encephalitis virus and NiV real-time RT-PCR assays were performed in singleplex using the following primers and minor groove-binding probes labeled with 6-carboxyfluorescein reporter dye and a nonfluorescent quencher: JEV-F: 5′-GGTGGACGGCCAGATTGAC, JEV-R: 5′-CCCCAARCATCAGCACAAG-3′, and JEV-P: 5′-CCTGCGGTTTTGGG-3′ and NiV-F: 5′-TGGAGCTGCTTTYACACTCATC-3′, NiV-R: 5′-TACAGCTTCAATGTCTGGGTCATT-3′, and NiV-P: 5′-TATGTATTCAGAGAGACCCGG-3’. All reagents were purchased from Applied Biosystems (Thermo Fisher).

### In-house PCR for the verification of *S. pneumoniae* and *H. influenzae*.

Samples with the detection of *S. pneumoniae* and/or *H. influenzae* in either of the multiplex PCR panels were further analyzed using an in-house real-time PCR^23^ to assess a semiquantitative measure of the amount of bacterial DNA. Samples extracted using Magna Pure Compact (Roche Diagnostics, Basel, Switzerland) were analyzed by a duplex LightCycler PCR assay (Roche Diagnostics) targeting the *lytA* and *P6* genes, respectively, and the results were assessed based on cycle threshold (Ct) value (Supplemental Material 1).

### Ethics.

Informed written consent was obtained before inclusion by the patient or from a guardian or caregiver if the patient was unable to provide informed written consent. The study protocol was ethically approved by the Nepal Health Research Council (Reg. No. 108/2014) and by the Regional Ethical Review Board in Uppsala, Sweden (reference number 2014/043).

## RESULTS

**Figure 2.**
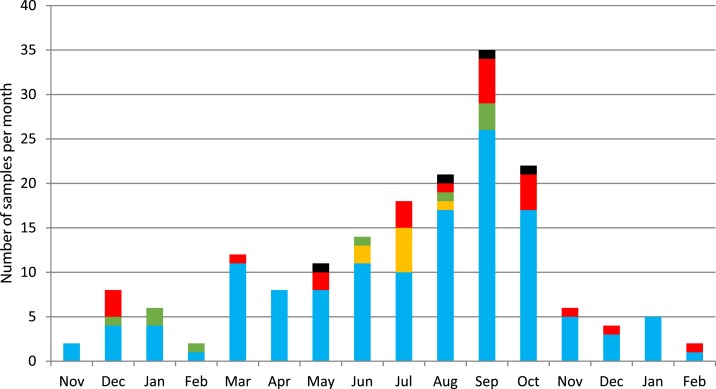
Number of collected samples from November 2014 to February 2016 presented per month, with the distribution of findings using molecular methods; enterovirus (orange), other viruses (red), bacteria (green), and *Cryptococcus* (black), and no detection (blue). This figure appears in color at www.ajtmh.org.

During the inclusion period, 176 clinical CSF samples from 174 patients were prospectively included for analysis. Of the samples, 65% were collected in June to October (monsoon season) (Figure 2). The median age was 13 years (range 0–85 years) and 55% were male (Table 2).

**Table 2:** Patient characteristics based on available questionnaire data

	No/available data (%)
Characteristics	
Males	61/111 (55)
Symptoms on admission	
Fever	44/61 (72)
Headache	38/58 (66)
Neck stiffness	34/63 (54)
Altered mental status	19/60 (32)
Rash	3/64 (5)

Of the patients, 97% came from Palpa and surrounding districts, which are characterized by a predominately hilly terrain, and 3% from lowland Terai area (Figure 1). Of the patients, 67% had been treated with antibiotics before arrival at the hospital; 72% of these had taken oral cephalosporins. Intravenous antibiotics were administered to 98% of the patients at some point during their hospital stay.

Onsite analysis showed 88% of the samples had at least 5 cells/µL, indicating inflammatory reaction within the CNS, possibly caused by infection (median 40 cells/µL, 10th percentile 2 cells/µL, and 90th percentile 420 cells/µL).

The most prevalent bacteria detected were *H. influenzae* (*n* = 5) and *S. pneumoniae* (*n* = 4), whereas *Neisseria meningitidis* was found in one sample*.* The most common virus was enterovirus detected in eight samples, all during the monsoon season from June to August (Figure 2). Other viruses detected were cytomegalovirus (CMV) (*n* = 6), varicella zoster virus (*n* = 5), EBV (*n* = 3), herpes simplex virus (HSV) type 1 (HSV-1) (*n* = 3), HSV type 2 (HSV-2) (*n* = 3), human herpes virus (HHV) type 6 (HHV-6) (*n* = 3), and HHV type 7 (HHV-7) (*n* = 2). *Cryptococcus neoformans/gatti* was found in four samples, two of these from patients with known HIV infection and the other two patients with unknown HIV status. *Borrelia burgdorferi* sensu lato, *Borrelia miyamatoi*, *Escherichia coli* K1, *Listeria monocytogenes*, *Streptococcus agalactiae*, DENV, HHV-8, human parechovirus (HPeV), JEV, morbillivirus, and NiV were not detected (Table 1). All samples with CSF WBC < 5 cells/µL were tested negative (Table 3). In eight samples, more than one pathogen was detected.

**Table 3:** Results of the polymerase chain reaction analyses in relation to CSF WBC count and discharge diagnosis

	No. of samples	Bacteria detected	Virus detected	Fungi detected	Pathogen not detected
CSF WBC count/µL					
0–4	15	0	0	0	15
5–99	74	5	8	2	60
100–999	35	3	13	1	18
≥ 1,000	5	0	2	0	3
Not recorded	47	1	6	1	40
Discharge diagnosis*					
Bacterial CNS infection	38	2	4	1	31
Viral CNS infection	7	0	4	0	3
Typhoid fever	5	0	0	0	3
TB	3	0	2†	0	1
Other, e.g., pneumonia	3	0	0	0	3
Not recorded	120	6	18	3	95

CNS = central nervous system; CSF = cerebrospinal fluid; TB = tuberculosis; WBC = white blood cell.

* Assessments of discharge diagnoses are based on clinical presentation, results from CSF WBC count analysis, and response to treatment.

† Cytomegalovirus and human herpes virus type 7 were detected in two patients with clinically diagnosed tuberculosis meningitis, suggesting viral reactivation induced by concomitant infection.

Enterovirus was detected in eight samples with the FilmArray ME panel. None of these were detected with the MeningoFinder 2SMART panel when using the limit of detection provided by the manufacturer. However, by manual assessment using a lower limit of detection, six out of these eight samples were assessed positive.

A similar result was found for the detection of *Cryptococcus*. Among the four samples where *Cryptococcus* was detected by the FilmArray ME panel, three were positive by MeningoFinder 2SMART at the limit of detection provided by the manufacturer. The fourth sample was positive in MeningoFinder 2SMART when a lower limit of detection was used.

*Haemophilus influenzae* and *S. pneumoniae* were detected in 58 and 48 samples, respectively, using the two multiplex PCR panels, FilmArray ME and MeningoFinder 2SMART panels. Because this number of detections was much higher than expected, all these samples were reanalyzed using a previously validated in-house PCR,^23^ which could confirm the presence of *H. influenzae* in 44 and *S. pneumoniae* in 34 samples tested. However, in most of the samples, the Ct values were very high, indicating very low quantities of DNA, and the detected DNA was, thus, considered as contamination rather than clinically relevant findings. The in-house PCR resulted in low Ct values, indicating high levels of DNA, for *H. influenzae* in five samples and *S. pneumoniae* in four samples (Supplemental Material 1). These samples were considered as true positives.

## DISCUSSION

To explore the complex etiology behind CNS infections, we prospectively investigated clinical samples, collected at a hospital serving a rural area in Nepal, using FilmArray ME and MeningoFinder 2SMART panels, and an in-house PCR for DENV, JEV, and NiV, which resulted in the identification of a likely etiological agent in 23% of the cases.

The most commonly detected bacteria were *S. pneumoniae* and *H. influenzae*, with detection rates in line with previous studies, whereas the detection of *N. meningitidis* was unexpectedly low.^24^

Enterovirus was the most commonly detected virus, with a clear relation to the monsoon season, in concordance with previous findings.^8^ This suggests that seasonal outbreaks of suspected viral AES in Nepal, at least in part, may be due to enterovirus infections. Enteroviruses are associated with a wide variety of clinical syndromes, including benign aseptic meningitis and acute flaccid paralysis/myelitis, the latter caused by polio or non-polio enteroviruses. Certain types of enteroviruses have been linked to outbreaks of CNS infections worldwide, including the neighboring countries of India and China.^25^ In this study, we did not aim to identify the specific enterovirus types responsible for infection.

In a number of samples, we detected viruses associated with severe diseases in primarily the immunocompromised patients. For example, CMV was detected in five patients of whom none had a diagnosed HIV infection or other known causes of immune suppression. These findings are hard to interpret as CMV is probably not a significant cause of acute CNS infections in non-immunocompromised patients. However, and importantly, from our clinical experience using the FilmArray ME Panel, the finding of CMV in CSF samples is very rare (data not shown). As with CMV, the detection of HHV-6 in a CSF sample can also be challenging to interpret as this virus can be chromosomally integrated or remain latent in mononuclear cells. However, the one patient with HHV-6 only presented with neonatal seizures, which is compatible with a primary HHV-6 infection.^26^

As the commercial multiplex PCR panels used in this study did not include JEV, DENV, or NiV, we added this analysis using an in-house PCR method. However, we did not detect any of these three viruses in this material. Nipah virus is an emerging zoonotic virus carried by bats with high case fatality rate in humans.^27^ Because of the epidemic potential of this virus, it is considered a global health priority by the World Health Organization, and outbreaks have been reported in Bangladesh and India.^27^ To the authors’ knowledge, this is the first study where clinical CSF samples in Nepal are tested for NiV.

Of the samples in this study, 77% were negative, despite the very broad panel of potential causative agents of CNS infection analyzed using sensitive methods. We find this figure to be relatively high, which might be explained by a number of causes. For example, bacterial meningitis is a rapidly progressive disease and some patients may not make it to the hospital in time because of the challenging terrain in the area. In addition, the high rate of self-treatment with antibiotics before admission may have reduced the chance of finding the disease-causing bacteria.^10^ However, we suggest that the most likely explanation is that the methods used in this study do not cover all the pathogens causing CNS infections in the current area.

In addition to the etiological agents investigated in the present study, CNS infections in Nepal could be caused by other viruses (e.g., chikungunya, rubella, West Nile, and rhabdoviruses) and bacteria (*Leptospira*, *Orientia tsutsugamushi*, and *Streptococcus suis*) as reported in studies from neighboring regions.^28,29^ Moreover, encephalopathy without direct CNS involvement could be due to malaria and enteric fever, diseases that are prevalent in Nepal.^30–32^ Tuberculosis (TB) is another important cause of serious CNS infections in Nepal, although the incidence is unknown.^33^ We choose not to include TB in our study protocol as the detection of *M. tuberculosis* with PCR requires a larger amount of CSF than was available. In this study, it is important to note that although no microbiological testing for TB was performed, we estimate that around 10 patients possibly could have had a TB infection based on the clinical picture, CSF cell count, and negative molecular testing for the named included pathogens.

Although several of the pathogens studied were not detected (*B. burgdorferi* sensu lato, *B. miyamatoi*, *E. coli* K1, *L. monocytogenes*, *S. agalactiae*, DENV, HHV-8, HPeV, JEV, morbillivirus, and NiV), this study cannot rule out the existence of these pathogens in the region. This is particularly important to notice in relation to DENV and JEV that are endemic in Nepal with a fluctuating incidence.^17,34^

Previous studies on the etiology of CNS infections in Nepal have reported *S. pneumoniae*, *H. influenzae*, *N. meningitidis*, *Staphylococcus aureus*, *S. agalactiae*, *E. coli*, *M. tuberculosis*, enteroviruses, HSV, and JEV as causative agents.^6–9,30^ In these studies, the etiology was still unclear in 62–93% of the patients. In Nepal and other parts of Asia, CNS infections are a major health concern. As this study together with several others could not find the causative pathogens in a majority of patients, by using conventional or molecular testing, this calls for a wider approach of the methods used, including metagenomic techniques.^35–37^

In this study, two-thirds of the patients had been exposed to antibiotics before lumbar puncture. Most of these patients had taken broad-spectrum antibiotics before arrival at the hospital, which may lead to delayed treatment, or, in the case of viral infections, may cause overuse of antibiotics, potentially leading to development of microbial resistance in the population.^38^ Also, nearly all patients in the study received intravenous antibiotics during some part of the hospital stay, as bacterial infections could not be excluded based on clinical signs and the laboratory tests available. This highlights the need for better diagnostic options for CNS infections in Nepal and elsewhere that can reliably exclude bacterial infections, to decrease inappropriate antibiotic use.

An unexpected large proportion of detections for *H. influenzae* and/or *S. pneumoniae* were seen using the FilmArray ME and MeningoFinder 2SMART panels. Because these tests present only a qualitative result, all samples positive for *H. influenzae* and/or *S. pneumoniae* were further analyzed with in-house real-time PCR to estimate the amount of bacterial DNA. This analysis confirmed the presence of DNA from these bacteria in most samples, but in most samples with signs of very low bacterial load. After thoroughly evaluating the issue, we considered those results to indicate contamination at some point of sample handling rather than clinical infection.

The strengths of this study include the prospective design with the consecutive inclusion of patients and that all samples were tested for a large number of pathogens.

Some limitations can be identified. First, clinical, demographic, and laboratory data were not completely recorded for all patients precluding detailed analysis of correlations between these data and the result from the molecular analysis. Second, only CSF samples were available for analysis and not serum/plasma samples for PCR and/or serology. Third, the analyses do not provide information about pathogen load, antimicrobial susceptibility, or further characterization of the pathogens, including serotypes and capsular types, emphasizing the need for additional testing in many cases.

To conclude, by using two commercial multiplex PCR panels combined with a specific PCR targeting three additional viruses, we could identify microorganisms capable of causing CNS infections in 23% of the samples. This low percentage indicates that the commercial methods are not suitable for use in the current setting and that future studies on the etiology of CNS infections in Nepal should include serum samples and may include a metagenomic approach.

## Supplementary Files

Supplemental materials
