# Supplementary material for: Etiology of Central Nervous System Infections in a Rural Area of Nepal Using Molecular Approaches
Source: Am J Trop Med Hyg. 2019 Jun 3;101(1):253–9. doi: 10.4269/ajtmh.18-0434 (PMC6609203; doi:10.4269/ajtmh.18-0434)
Supplement: Supplementary file 1 [file tpmd180434.SD1.pdf]

Supplementary material 1.

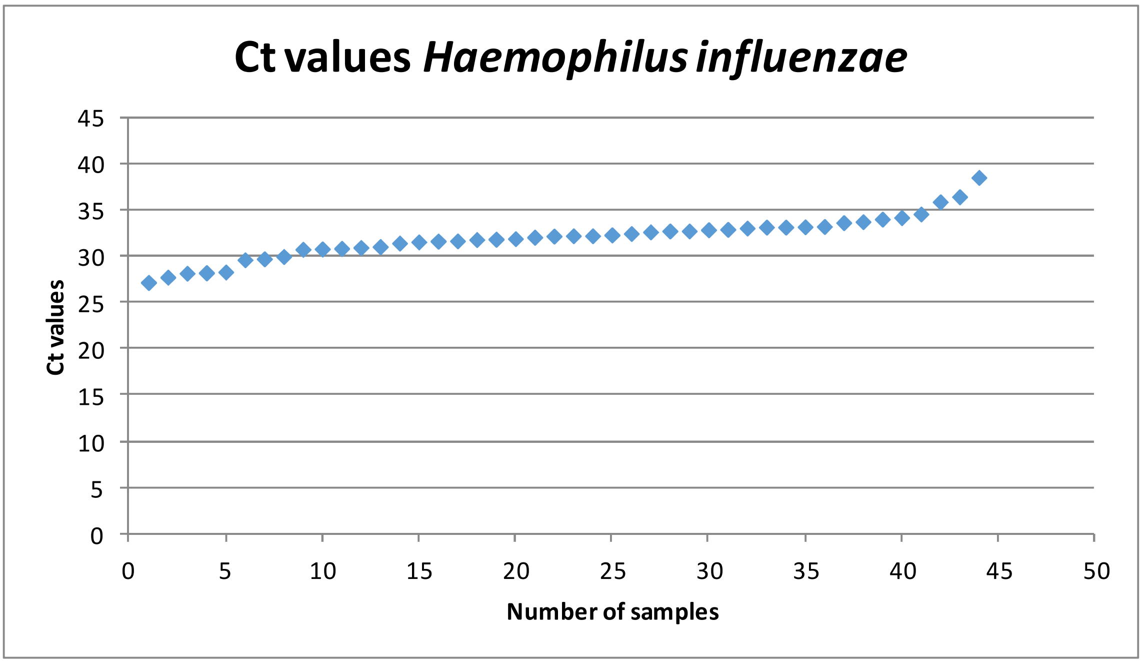


All samples with detections of *H. influenzae* in FilmArray® ME Panel and/or MeningoFinder® 2SMART (n=58) were further analyzed using in-house PCR, which could confirm the presence of *H. influenzae* in 44 samples. In 5 of these samples, the Ct values were low, indicating high levels of DNA. These samples were considered as true positives.

| Ct value *H. influenzae* | FilmArray® ME Panel positive MeningoFinder® 2SMART positive | FilmArray® ME Panel positive MeningoFinder® 2SMART negative | FilmArray® ME Panel negative MeningoFinder® 2SMART positive | Both panels negative |
|---|---|---|---|---|
| <29.00 | 4 | 1 | | |
| 29.00-34.00 | 15 | 16 | 1 | |
| >34.00 | 2 | 1 | | 3 |

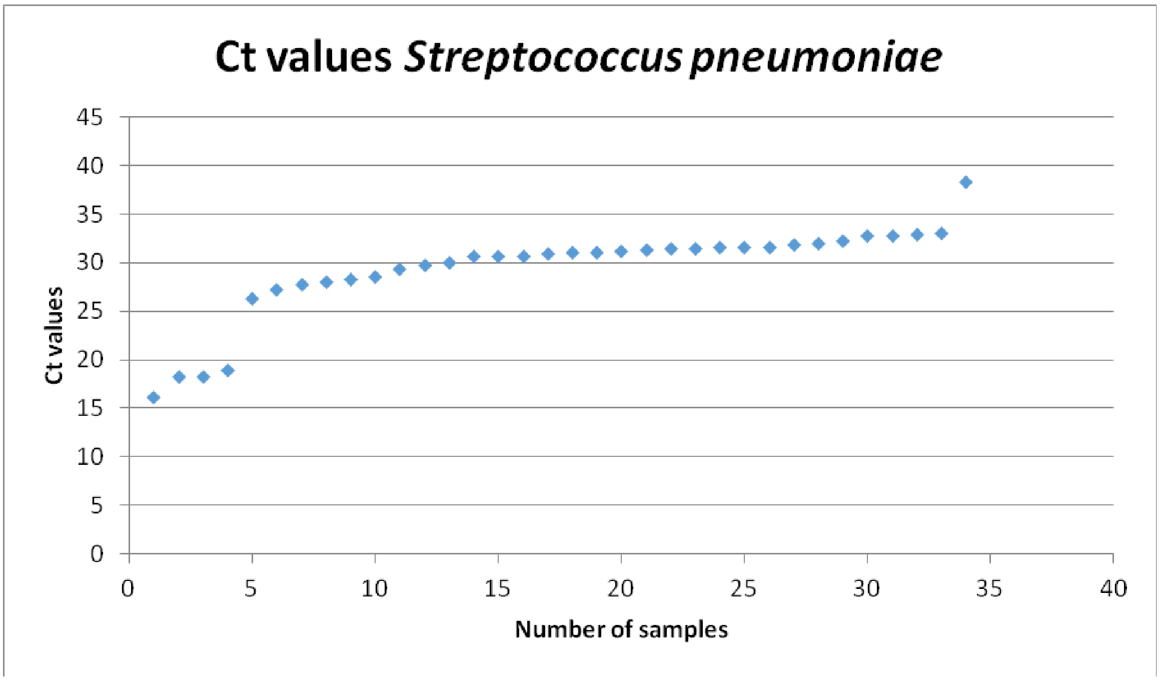


All samples with detections of *S. pneumoniae* in FilmArray® ME Panel and/or MeningoFinder® 2SMART (n=48) were further analyzed using in-house PCR, which could confirm the presence of *S. pneumoniae* in 34 samples.  In 4 of these samples, the Ct values were low, indicating high levels of DNA. These samples were considered as true positives.

| Ct value *S. pneumoniae* | FilmArray® ME Panel positive MeningoFinder® 2SMART positive | FilmArray® ME Panel positive MeningoFinder® 2SMART negative | FilmArray® ME Panel negative MeningoFinder® 2SMART positive | Both panels negative |
|---|---|---|---|---|
| <20.00 | 4 | | | |
| 20.00-30.00 | 7 | 1 | | |
| 30.01-31.99 | 11 | 3 | | |
| >32.00 | 2 | 4 | | 1 |
